# Y_2_O_3_NPs induce selective cytotoxicity, genomic instability, oxidative stress and ROS mediated mitochondrial apoptosis in human epidermoid skin A-431 Cancer cells

**DOI:** 10.1038/s41598-024-82376-w

**Published:** 2025-01-09

**Authors:** Hanan RH Mohamed, Shrouk H.A Hemdan, Ahmed A. El-Sherif

**Affiliations:** 1 Department of Zoology, Faculty of Science, Cairo University, Giza, Egypt; 2 Department of Chemistry, Faculty of Science, Cairo University, Giza, Egypt

**Keywords:** Yttrium oxide nanoparticles, Cytotoxicity, genotoxicity, apoptosis, Oxidative stress and mitochondria, Skin cancer, Cell biology, Molecular biology, Medical research

## Abstract

Yttrium oxide nanoparticles (Y_2_O_3_NPs) have emerged as a promising avenue for cancer therapy, primarily due to their distinctive properties that facilitate selective targeting of cancer cells. Despite their potential, the therapeutic effects of Y_2_O_3_NPs on human epidermoid skin cancer remain largely unexplored. This study was thus conducted to investigate the impact of Y_2_O_3_NPs on both human skin normal and cancer cells, with an emphasis on assessing their cytotoxicity, genotoxicity, and the mechanisms underlying these effects. Cell viability and apoptosis induction were assessed using the Sulforhodamine B and chromatin diffusion assay, respectively. Reactive oxygen species (ROS) level, mitochondrial membrane potential integrity, oxidative stress markers and expression level of apoptotic and mitochondrial genes were also estimated. Our findings highlight the selective and significant cytotoxicity of Y_2_O_3_NPs against human epidermoid A-431 cancer cells. Notably, exposure to five Y_2_O_3_NPs concentrations (0.1, 1, 10, 100 and 1000 µg/ml) resulted in a high concentration-dependent reduction in cell viability and a corresponding increase in cell death observed 72 h post-treatment specifically in A-431 cancer cells, while normal skin fibroblast (HSF) cells exhibited minimal toxicity. When A-431 cancer cells were treated with the half-maximal inhibitory concentration (IC50) of Y_2_O_3_NPs for 72 h, a significant increase in ROS generation was noted. This led to oxidative stress, along with severe damage to genomic DNA and mitochondrial membrane potential, triggering substantial apoptosis. Furthermore, a concurrent significant upregulation of apoptotic p53 and mitochondrial ND3 genes was observed, coupled with a notable decrease in the anti-apoptotic Bcl2 gene expression.

Overall, Y_2_O_3_NPs demonstrate considerable promise as a therapeutic agent for skin epidermoid cancer due to their ability to selectively target and induce cytotoxic effects in A-431 cancer cells, all while causing minimal harm to normal HSF cells. This selective cytotoxicity appears to be associated with Y_2_O_3_NPs’ ability to induce excessive ROS production and subsequent oxidative stress, leading to significant genomic DNA fragmentation, loss of mitochondrial permeability, and alterations in apoptotic and mitochondrial genes’ expression, ultimately promoting apoptosis in A-431 cancer cells. These findings establish a foundation for further research into the utilization of Y_2_O_3_NPs in targeted cancer therapies and underscore the necessity for ongoing investigation into their safety and efficacy in clinical applications.

## Introduction

Skin cancer is one of the most prevalent forms of cancer globally, with a significant impact on public health due to its high incidence and associated morbidity. Among the various types of skin cancer, epidermoid skin cancer, or squamous cell carcinoma, is particularly common. Epidermoid skin cancer originates from the squamous cells in the epidermis, the outermost layer of the skin, and is primarily driven by chronic exposure to ultraviolet radiation^1^. Epidermoid skin cancer often presents as a scaly, red patch or a sore that does not heal, and can sometimes be mistaken for other skin conditions^2^. It is frequently observed in sun-exposed areas of the skin, making individuals with a history of excessive sun exposure or sunburn particularly susceptible^3^.

While epidermoid skin cancer is generally less aggressive than melanoma, it can still lead to significant morbidity and, in rare cases, metastasis if left untreated^4^. Effective management of epidermoid skin cancer requires a comprehensive approach, including early detection, accurate diagnosis, and appropriate treatment. Advances in therapies, including novel approaches like nano-therapy, are enhancing treatment outcomes and providing new options for patients^5^.

In light of these challenges, recent advances in nanotechnology are emerging as promising avenues for both diagnosis and treatment. Notably, yttrium oxide nanoparticles (Y_2_O_3_NPs) are gaining attention as potential agents in medical applications, including imaging and targeted therapy^6^. These nanoparticles are not only valuable in the medical field but are also utilized across various sectors such as electronics and optics, showcasing their versatile physical and chemical properties. From enhancing LED lighting to serving as contrast agents in imaging technologies, Y_2_O_3_NPs demonstrate superior performance in comparison to their bulk counterparts, thanks to their unique size and surface characteristics^7^.

Research into the efficacy of Y_2_O_3_NPs has garnered attention for their targeted cytotoxicity and genotoxicity against human cancer cells. Notably, recent studies demonstrated that exposure to Y_2_O_3_NPs results in a concentration-dependent reduction in the viability of human triple-negative breast and prostate cancer cells. This effect is primarily attributed to the generation of excessive reactive oxygen species (ROS), which ultimately disrupts mitochondrial membrane integrity, induces DNA damage and oxidative stress triggering apoptosis. In contrast, normal human cells, such as Human Skin Fibroblasts (HSF) and Human Dermal Fibroblasts (HDF), have demonstrated resilience, with no significant alterations in cell viability or DNA integrity following treatment with Y_2_O_3_NPs. Despite these promising results, there remains a notable lack of research exploring the impact of Y_2_O_3_NPs on melanoma cells^8,9^.

Given this gap in knowledge, our study was thus done to estimate the effects of Y_2_O_3_NPs on the viability of epidermoid skin cancer A-431 cells alongside normal HSF cells. The influence of Y_2_O_3_NPs on genomic DNA integrity, mitochondrial integrity, and induction of oxidative stress and apoptosis in human skin epidermoid A-431 cancer cells were also assessed. The Sulforhodamine B (SRB) assay for cell viability evaluation, while, apoptosis induction was detected using chromatin diffusion assay. The mitochondrial membrane potential integrity, ROS generation level, the expression of apoptotic and mitochondrial and oxidative stress markers were also assessed.

## Materials and methods

### Chemicals

The Y_2_O_3_NPs utilized in this study were acquired from Sigma-Aldrich Company (St. Louis, MO, USA), and are cataloged under product number 544,892. These nanoparticles were the same nanoparticles previously used by Emad et al.^9^. , and were provided in the form of white powders, with a particle size of less than 50 nm and a purity of 99.9% with respect to trace metals. All other materials and supplies employed throughout the experimental procedures were sourced at high molecular grade to ensure consistency and accuracy in our methodologies.

## Characterization of Y2O3NPs

To characterize Y2O3NPs, X-ray diffraction (XRD) analysis was performed to estimate the crystal structure and purity of the purchased Y_2_O_3_NPs powders. Dynamic laser scattering (DLS) was also performed to evaluate the stability and distribution of the suspended Y_2_O_3_NPs particles. Furthermore, Y_2_O_3_NPs were imaged using transmission electron microscopy (TEM) to reveal the morphology and average particle size of the suspended Y_2_O_3_NPs particles.

## Cell lines

Human epidermoid skin cancer A-431 and normal skin fibroblast (HSF) cell lines were purchased from Nawah Scientific Inc. (Mokatam, Cairo, Egypt). Normal HSF and cancerous A-431 cells were separately cultured in DMEM medium supplemented with 10% inactivated fetal bovine serum, 100 units/mL penicillin, and 100 mg/mL streptomycin. The cultured cells were maintained in an incubator at 37 °C with 5% CO2.

## Cell viability

To estimate the influence of Y_2_O_3_NPs exposure on viability of normal HSF and cancerous A-431 cells, Sulforhodamine B (SRB) assay was conducted^10,11^. Briefly, a 100 µL normal HSF and cancerous A-431 cells (100 ) cell suspension were separately cultured in 96-well plates and incubated for 24 h in complete media. After incubation, HSF and A-431 cells were treated with five different concentrations of Y_2_O_3_NPs (0.1, 1, 10, 100–1000 µg/mL) in accordance with the study of Mohamed et al.^12^, and left for 72-hour. The treated cells with Y_2_O_3_NPs were then fixed, washed with distilled water, mixed with SRB solution (0.4% w/v) and left for 10 min at room temperature in the dark. After that, plates of treated HSF and A-431 cells were washed with acetic acid (1%) and left to dry overnight. For analysis, the protein-bound SRB stain was dissolved, and the absorbance was measured at 540 nm using a BMG LABTECH^®^-FLUO Star Omega microplate reader (Ortenberg, Germany). GraphPad Prism software was employed to determine the half-maximal inhibitory concentration (IC50) of the three treated replicates. Selectivity index (SI) was calculated using the following formula:

SI = IC50 in normal cells/IC50 in tumor cells.

## Cell treatment

Human epidermoid skin cancer A-431 cells were seeded in T25 flasks under appropriate conditions, these cells were then divided into untreated (control) and doxorubicin- or Y_2_O_3_NPs-treated cells. The untreated control cells were exposed to DMSO at a concentration less than 0.1%, while treated A-431 cells were treated with doxorubicin (2µM/ml) or Y_2_O_3_NPs at a concentration equal to ¼ IC50, ½ IC50 or IC50 value for 72 h. After treatment, control and treated A-431 cells were harvested, washed twice with ice-cold PBS and stored at -80 °C in PBS for further molecular analysis. Triplicate was conducted for control and treated A-431 cells to ensure accuracy and consistency in the results.

### Estimation of apoptosis induction

The induction of apoptosis in both control and Y_2_O_3_NPs -treated A-431 cells was assessed using a chromatin diffusion assay based on the principle that apoptotic cells exhibit numerous alkali-labile sites, which, when exposed to alkaline conditions, yield small fragments of DNA. These DNA fragments can readily diffuse within an agarose matrix, manifesting as a halo with a hazy outline^13^. In brief: after coating microgel electrophoresis slides with a layer of agarose (0.7%), a mixture of A-431 cells and agarose was carefully placed on these pre-coated slides and spread evenly. The slides were allowed to air dry to solidify the agarose and encapsulate the cells. After drying, the slides were immersed in a lysis solution for 10 min to lysis all cellular components except DNA. Following lysis, the slides were neutralized using a freshly prepared Tris buffer and fixed in absolute ethanol to stabilize the DNA structures. Prior analysis, the slides were stained with ethidium bromide to visualize DNA under a fluorescent microscope. Cells with diffuse DNA halos were classified as apoptotic, and the percentage of A-431 cells with diffuse DNA and a hazy outline was calculated based on a total count of 1000 cells.

## Estimation of ROS generation level

The effect of Y_2_O_3_NPs on ROS generation in cancerous A-431 cells was investigated using the 2,7-dichlorofluorescin diacetate (DCFH-DA) dye^14^. Cancerous A-431 cells were mixed with a 20 mM solution of DCFH-DA dye and incubated in the dark at room temperature for 30 min. The dye penetrates the cells and reacts with ROS to produce a fluorescent product called dichlorofluorescein. After incubation, the cell-dye mixture was placed on a slide, examined, and photographed under an epi-fluorescent microscope at 200X magnification to detect the fluorescent signal that indicates ROS production in the A-431 cells. The intensity of emitted fluorescent light was measured using **ImageJ/FIJI** free software.

## Biochemical measurement of oxidative stress markers

The level of lipid peroxidation product malondialdehyde (MDA) was determined using the reaction of MDA with thiobarbituric acid (TBA) in an acidic medium at a temperature of 95 °C. This reaction results in the formation of a TBA reactive product, as detailed by Ohkawa et al.^15^. The absorbance of the resultant pink product was measured using a spectrophotometer at a wavelength of 534 nm and results were expressed in mmol/ml. The activity of the antioxidant enzymes superoxide dismutase (SOD) and catalase (CAT) was measured following the protocols established by Nishikimi et al.^16^and Aebi^17^ respectively. The activity of SOD enzyme was assessed based on its ability to inhibit the phenazine methosulfate-mediated reduction of nitroblue tetrazolium dye, while, the activity of CAT was determined by the reaction of CAT with a known quantity of hydrogen peroxide (H_2_O_2_) and results of both SOD and CAT activities were expressed in units per milliliter U/ml.

### Detection of mitochondrial membrane potential integrity

The impact of Y_2_O_3_NPs on integrity of mitochondrial membrane potential was also studied using Rhodamine-123 fluorescent dye^18^. Cancerous A-431 cells were mixed with 10 mg/ml Rhodamine-123 dye and incubated for 1 h at 37 °C in the dark. The cells were then washed twice with PBS and spread on clean sterile slides. The fluorescence emitted by Rhodamine-123 was visualized and captured with an epi-fluorescence microscope at 200x magnification thus any changes in integrity of mitochondrial membrane potential upon exposure to Y_2_O_3_NPs could be assessed. The free software ImageJ/FIJI was used to analyze the captured photos and to measure the intensity of emitted fluorescent light.

### Measuring the mRNA expression level of apoptotic and mitochondrial genes

To measure the mRNA expression level of apoptotic (p53 and Bcl2) and mitochondrial NADH dehydrogenase 3 (ND3) genes in the control and Y_2_O_3_NPs -treated A-431 cancer cells using qRT-PCR, whole RNA was first extracted from A-431 cancer cells using the GeneJET RNA Purification Kit from Thermo Fisher Scientific Company (USA), and and then the extracted RNA was converted to complementary DNA (cDNA) using the cDNA Reverse Transcription Kit from Applied Biosystems (Foster City, CA, USA). Finally, qRT-PCR was performed to amplify and measure the expression level of p53, Bcl2, and ND3 genes in StepOnePlus Real-Time PCR System (Applied Biosystems) using SYBER Green PCR Master Mix (Applied Biosystems, USA) and primers^19–21^ listed in Table 1. The expression level of the amplified genes was standardized against the housekeeping GAPDH gene and calculated using the comparative Ct (ΔΔCt) method. Results were reported as mean ± SD.

**Table 1:** Sequences of primers used in qRT-PCR.

Gene	Strand	Primer’s sequences
**ND3**	**Forward**	**5’-CGCCGCCTGATACTGGCAT-3’**
	**Reverse**	**5’-CTAGTATTCCTAGAAGTGAG-3’**
**BCL-2**	**Forward**	**5’-TCCGATCAGGAAGGCTAGAGT-3’**
	**Reverse**	**5’-TCGGTCTCCTAAAAGCAGGC-3’**
**P53**	**Forward**	**5’-CAGCCAAGTCTGTGACTTGCACGTAC-3’**
	**Reverse**	**5’-CTATGTCGAAAAGTGTTTCTGTCATC-3’**
**GAPDH**	**Forward**	**5’-GAAGGTGAAGGTCGGAGTCA-3’**
	**Reverse**	**5’-GAAGATGGTGATGGGATTTC-3’**

### Statistical analysis

The results of alkaline Comet assay, ROS generation, integrity of mitochondrial membrane potential, qRT-PCR and oxidative stress markers were expressed as mean ± SD and were statistically analyzed using the Statistical Package for the Social Sciences 20 (SPSS 20). One-way analysis of variance (ANOVA) followed by Duncan’ test was done to compare untreated control and Y2O3NPs-treated cells. Regression and correlation analysis was also conducted to estimate the effect of different concentrations of Y2O3-NPs on genomic DNA integrity and genes’ expression in A-431 cancer cells.

## Results

### Y2O3NPs characterization

**Fig. 1:**
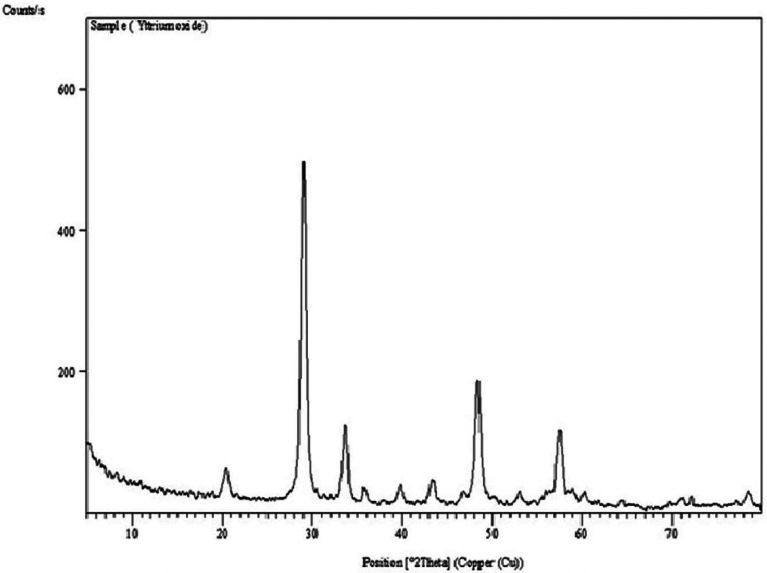
X-ray diffraction (XRD) pattern detected using X-Ray Diffractometers confirmed the purity and crystalline structure of purchased Y 2 O 3 NPs

**Fig. 2:**
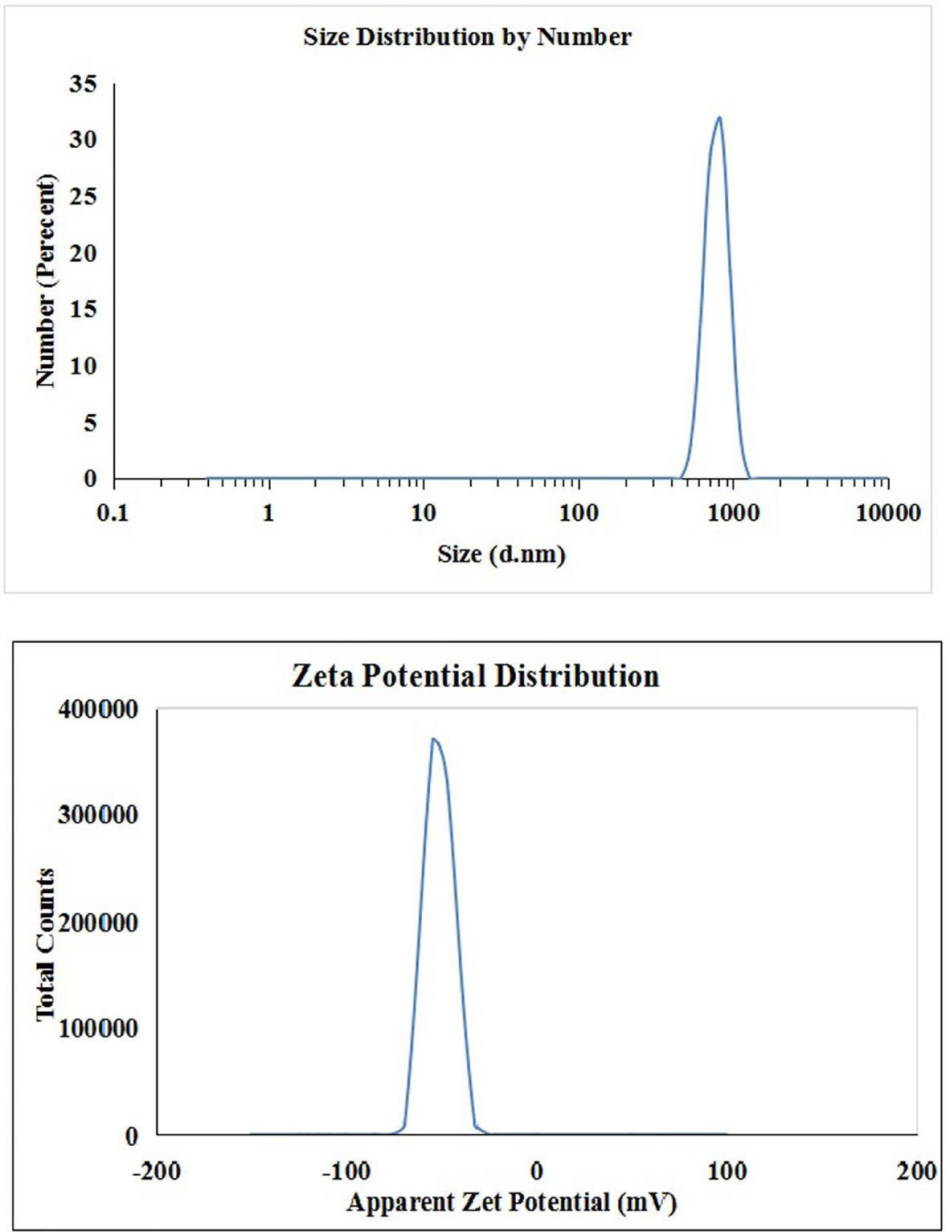
Particles’ Size Distribution and Zeta Potential Distribution of Y 2 O 3 NPs measured using Zetasizer revealed well dispersion and stability of suspended Y 2 O 3 NPs.

**Fig. 3:**
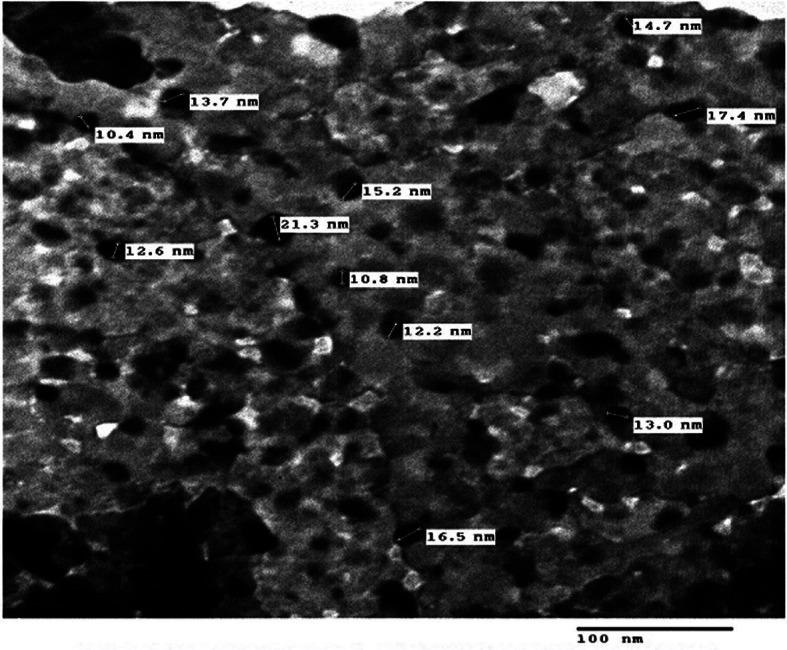
Photomicrograph of TEM imaging showed the well distribution and cubic shape of Y 2 O 3 NPs with an average particle size of 14.15 nm.

The results of XRD analysis, as illustrated in Fig. 1, confirmed the purity of Y_2_O_3_NPs utilized in this study. Characteristic peaks were observed at theta angles of 20.45º, 29.08º, 33.70º, 48.41º, and 57.47º, indicative of the Y_2_O_3_NPs in harmony with the study of Emad et al.^9^. Complementing these findings, DLS analysis demonstrated high aggregation of Y_2_O_3_NPs as evidenced by an average particle size of 792.61 nm for the dispersed Y_2_O_3_NPs alongside the Zeta potential value for Y_2_O_3_NPs was recorded at -54.12 mV (Fig. 2) highlighting the necessity for ultra-sonication to enhance the suspension of Y_2_O_3_NPs prior treatment. Imaging using TEM revealed a cubic shape and well dispersion of the ultra-sonicated Y_2_O_3_NPs in aqueous medium with a reduced mean particle’ size of 14.15 nm as depicted in Fig. 3.

**Fig. a:**
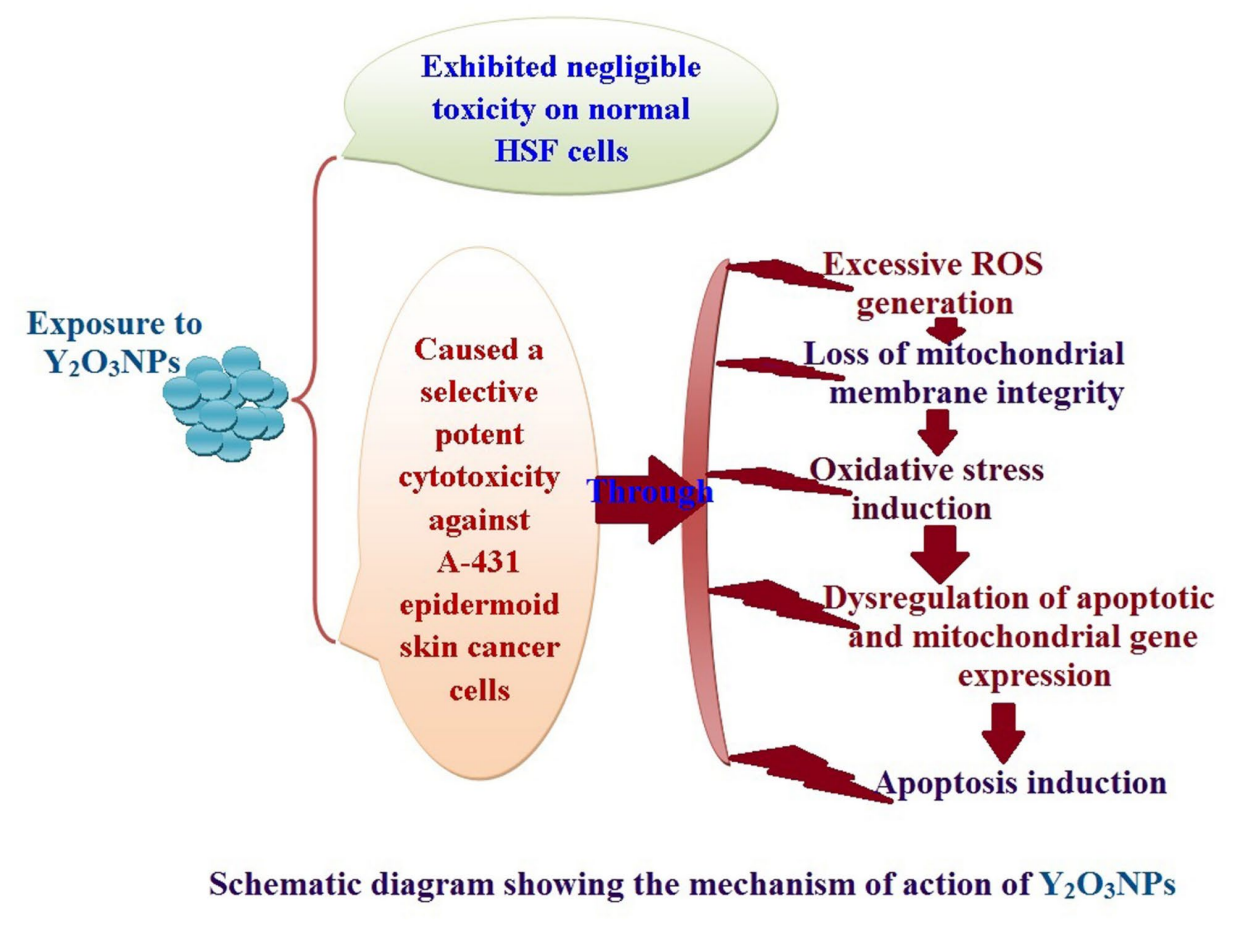
a

### Cell viability

**Fig. 4:**
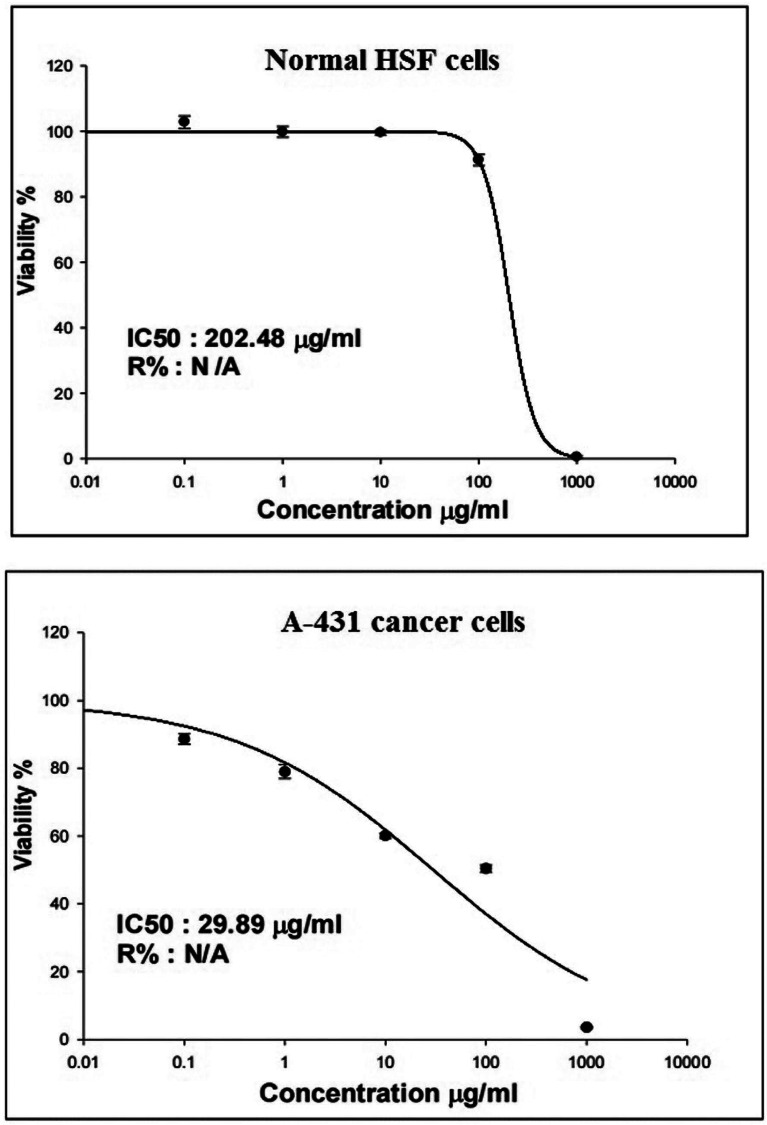
Viability of normal HSF and cancerous A-431 cells detected using SRB assay after exposure to five different concentrations of Y 2 O 3 NPs (0.1, 1, 10, 100 and 1000 µg/ml) for 72 h.

Screening viability of normal HSF cells and cancerous A-431 cells using SRB demonstrated a clear differential response to varying concentrations of Y_2_O_3_NPs. Cancerous A-431 cancer cells exhibited a significant concentration-dependent inhibition of proliferation and increased cell death after a 72-hour exposure to Y_2_O_3_NPs five tested concentrations (0.1, 1, 10, 100, and 1000 µg/ml). The calculated half-maximal inhibitory concentration (IC50) for A-431 cancer cells was determined to be 29.89 µg /ml as illustrated in Fig. 4. In contrast, the normal HSF cells showed only minimal changes in viability following treatment with Y_2_O_3_NPs at concentrations of 0.1, 1, 10, and 100 µg /ml. A significant increase in cell death among normal HSF cells was only observed at the highest concentration of 1000 µg/ml, with an IC50 value of 202.48 µg /ml (Fig. 4). The selectivity index for Y_2_O_3_NPs was calculated to be 14.76, indicating a high level of selective cytotoxicity towards A-431 cancer cells compared to normal HSF cells.

### Apoptosis induction in A-431 cells

**Fig. 5:**
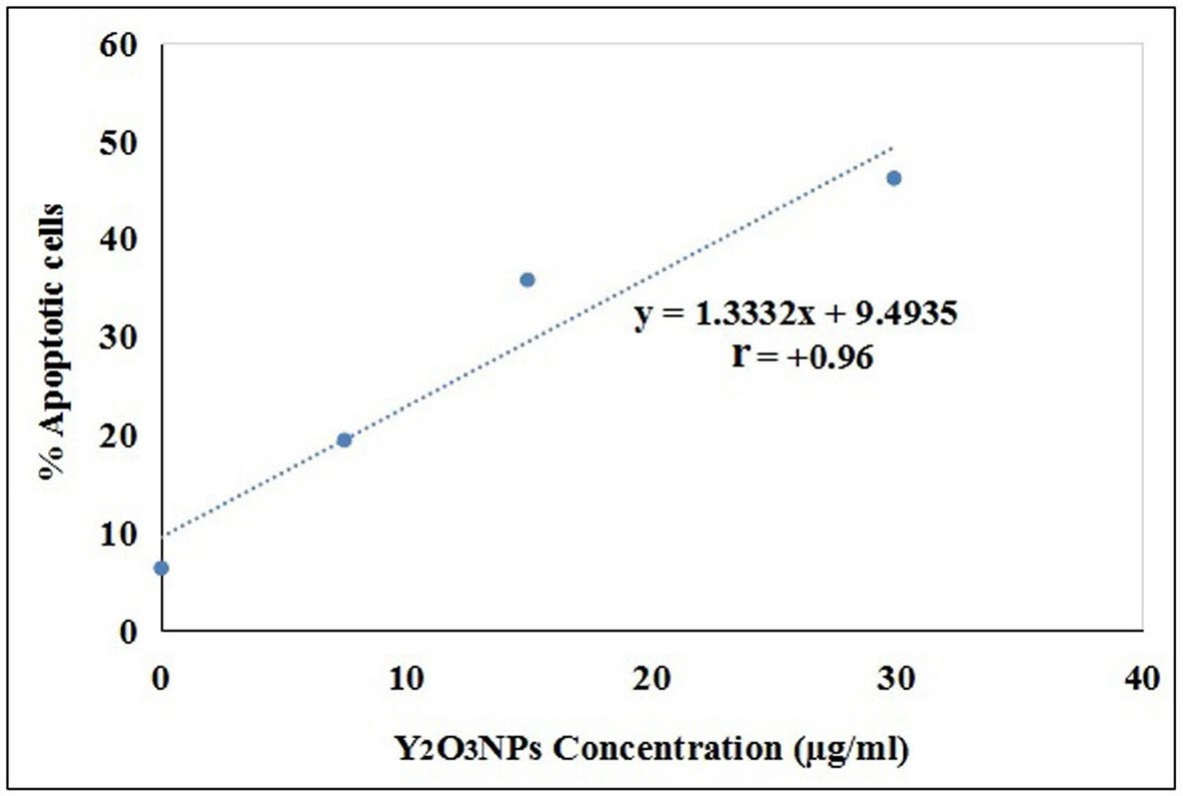
Regression line and correlation coefficient between Y 2 O 3 NPs different concentrations (7.47. 14.94 and 28.89 µg/ml) and the assessed %A-431 cells with diffused damaged DNA using Chromatin Diffusion assay.

**Fig. 6:**
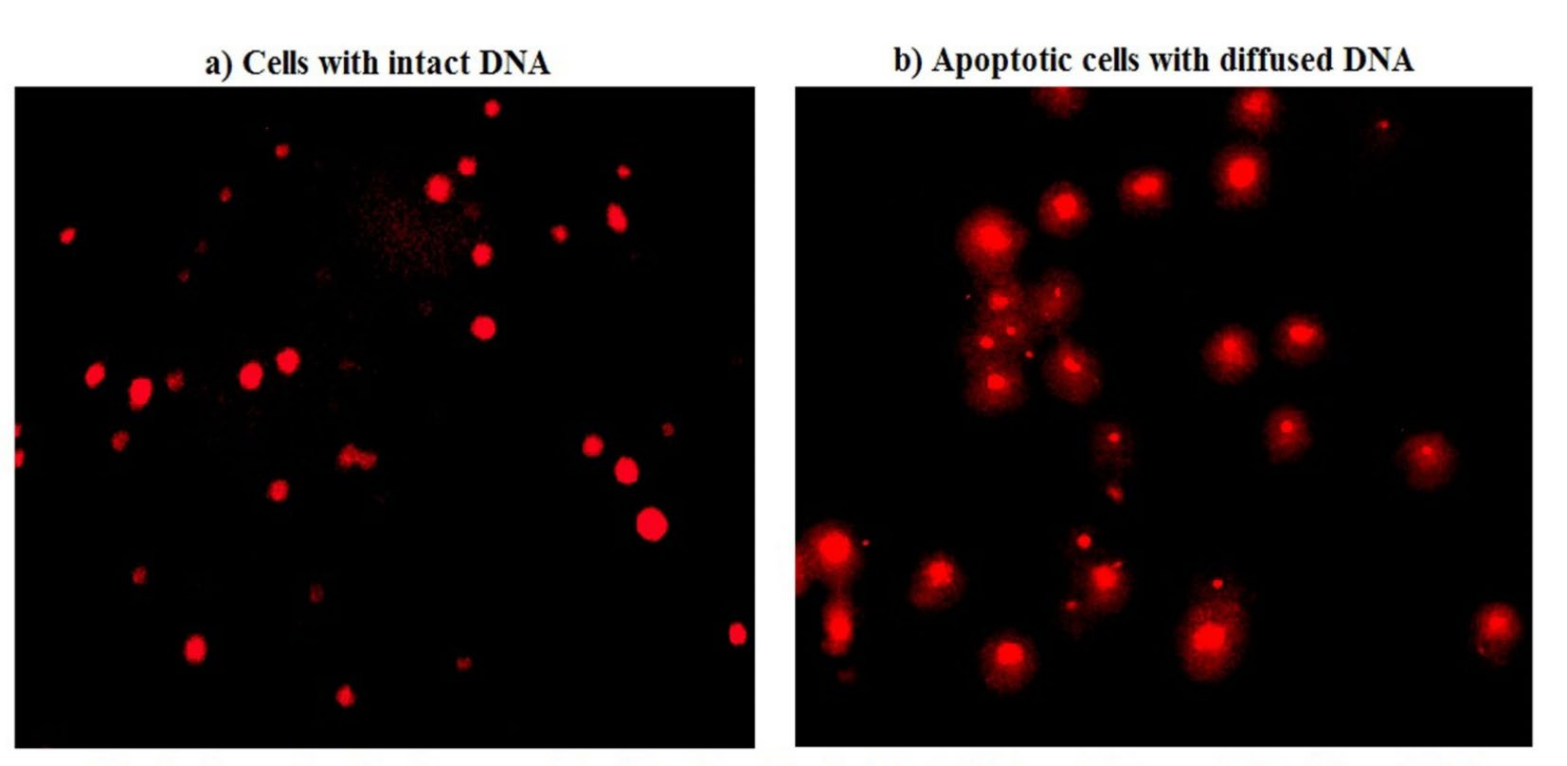
Examples for the scored A-431 cells with (a) intact DNA and (b) diffused DNA regardless treatment using Chromatin Diffusion assay.

Interpretation of chromatin diffusion assay results demonstrated a marked concentration-dependent induction of apoptosis in A-431 cancer cells upon exposure to three concentrations of Y_2_O_3_NPs (7.47, 14.94 and 29.89 µg/ml) as manifested by the concentration-dependent statistically significant elevations in the number of A-431 cancer cells with diffused DNA and % Apoptotic A-431 cancer cells compared to their values in the negative control and even doxorubicin A-431 cancer cells (Table 2). A strong positive correlation between the three tested concentrations of Y_2_O_3_NPs and % A-431 cancer cells with diffused DNA was also confirmed the high r value of + 0.96 obtained from the regression analysis curve as shown in Fig. 5. Moreover, representative examples of the scored A-431 cancer cells with intact and diffused DNA (apoptotic cells were shown in Fig. 6.

**Table 2:** Induction of apoptosis in the untreated control cells and cells treated with doxorubicin and Y_2_O_3_NPs different concentrations.

Treatment	Concentration (µg/ml)	No. of cells with intact DNA	No. of A-431 cells with diffused DNA	%Apoptotic cells
**Untreated cells**	**0.00 µg/ml**	**936.67 ± 4.04** ^a^	**63.33 ± 4.04** ^a^	**6.33 ± 0.40** ^a^
**Doxorubicin**	**2 µg/ml**	**781.67 ± 7.64** ^b^	**218.33 ± 7.64** ^b^	**21.83 ± 0.76** ^b^
**Y**_**2**_ **O**_**3**_ **NPs**	**1/4 IC50** **(7.47 µg/ml)**	**805.67 ± 5.03** ^**c**^	**194.33 ± 5.03** ^**c**^	**19.43 ± 0.50** ^**c**^
	**1/2 IC50** **(14.94 µg/ml)**	**642.33 ± 8.02** ^**d**^	**357.67 ± 8.03** ^**d**^	**35.77 ± 0.80** ^**d**^
	**IC50** **(29.89 µg/ml)**	**541.33 ± 10.26** ^**e**^	**461.67 ± 10.41** ^**e**^	**46.17 ± 1.04** ^**e**^
**ANOVA**		**F = 1303.30 < 0.001** *p*	**F = 1305.58 < 0.001** *p*	**F = 1305.58 < 0.001** *p*

• **Results are expressed as mean ± SD**.

• **Results were analyzed using One Way Analysis of Variance (ANOVA) followed by Duncan’s test.**

• **Different superscript letters indicate statistical significant difference among the compared untreated control and treated cells at < 0.001.***p*

• **F value is the ratio of between and within group variances**.

### ROS generation within A-431 cells

**Fig. 7:**
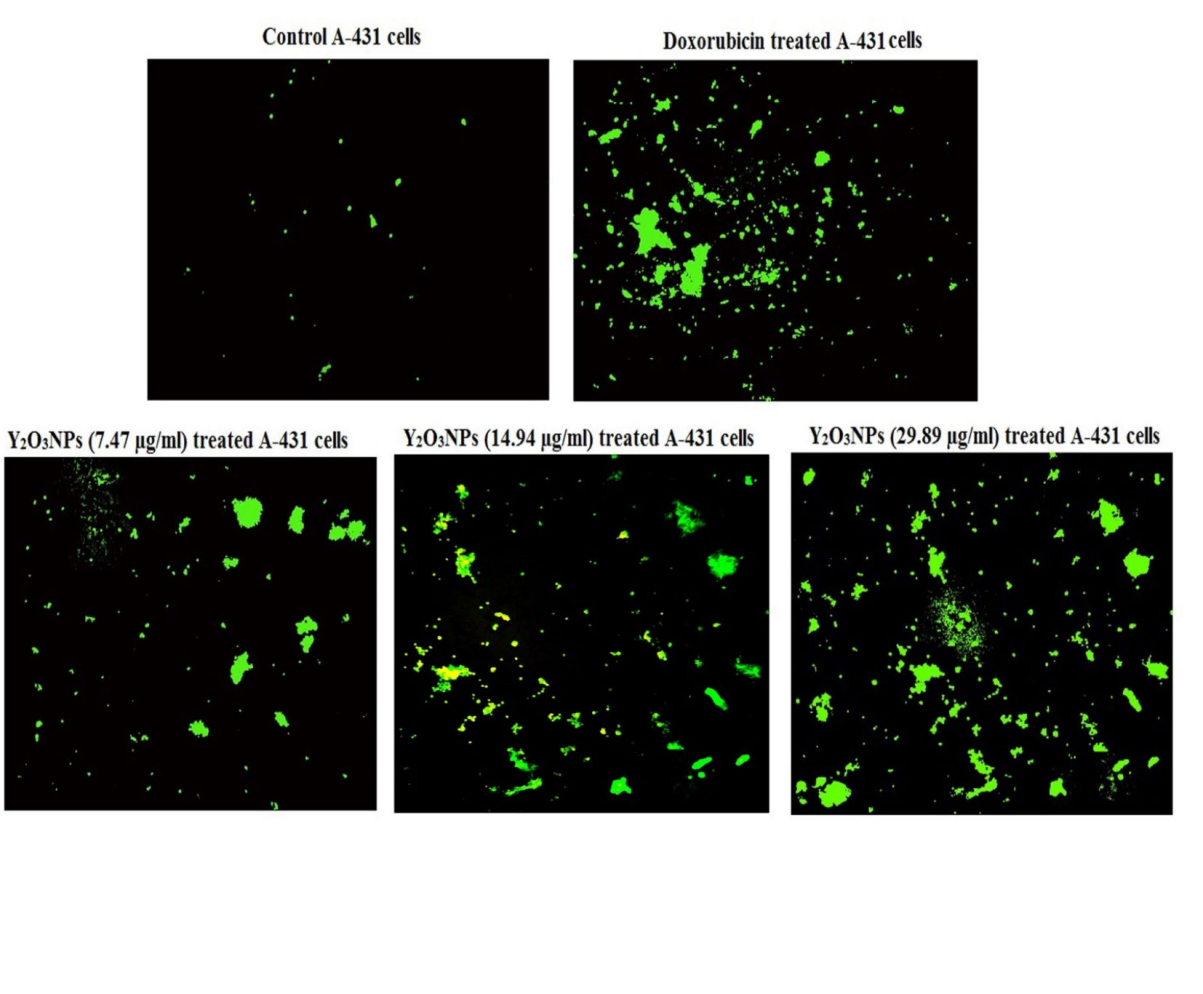
Level of ROS generation analyzed using 2 , 7 DCFH-DA dye within control untreated A-431 cells and A-431 cells treated with doxorubicin- or Y **2** O **3** NPs (7.47. 14.94 and 28.89 µg/ml) for 72 h .

Examination of 2,7-DCFH-DA stained A-431 cancer cells under fluorescent microscopy revealed a concentration-dependent marked increase in ROS production 72 h of A-431 cancer cells exposure to Y_2_O_3_NPs at concentrations of 7.47, 14.98 and 29.89 µg/ml compared to untreated (control) A-431 cells and doxorubicin-treated A-431 cancer cells as illustrated in Fig. 7; Table 3 through the remarkable elevations in the intensity of fluorescent light emitted from Y_2_O_3_NPs-treated A-431 cancer cells compared to the light emitted from both doxorubicin-treated A-431 cancer cells and untreated control A-431 cancer cells.

**Table 3:** Intensity of fluorescence emission by the 2,7-DCFH-DA dye (ROS level) or Rhodamine-123 dye (mitochondrial membrane integrity) stained control (untreated) and doxorubicin- or Y_2_O_3_NPs-treated A-431 cancer cells.

**Treatment**	**Concentration (µg/ml)**	**Intensity of fluorescence emission**	
		**ROS generation level**	**Mitochondrial membrane potential integrity**
**Control cells**	**0.00 µg/ml**	**1.28 ± 5.52** ^a^	**2.92 ± 15.25** ^a^
**Doxorubicin**	**2 µg/ml**	**6.87 ± 24.64** ^**b**^	**0.561 ± 6.45** ^**b**^
**Y**_**2**_ **O**_**3**_ **NPs**	**1/4 IC50** **(7.47 µg/ml)**	**4.46 ± 19.02** ^**c**^	**0.891 ± 8.41** ^**c**^
	**1/2 IC50** **(14.94 µg/ml)**	**4.68 ± 20.73** ^**c**^	**0.507 ± 6.07** ^**b**^
	**IC50** **(29.89 µg/ml)**	**7.74 ± 28.97** ^**d**^	**0.229 ± 4.13** ^**d**^
**ANOVA**		*p* < 0.001	*p* < 0.001

• **Results are expressed as mean ± SD**.

• **Results were analyzed using One Way Analysis of Variance (ANOVA) followed by Duncan’s test.**

• **Different superscript letters indicates statistical significant difference among the compared untreated control and treated cells at < 0.001.***p*

• **F value is the ratio of between and within group variances**.

### Induction of oxidative stress within A-431 cells

**Fig. 8:**
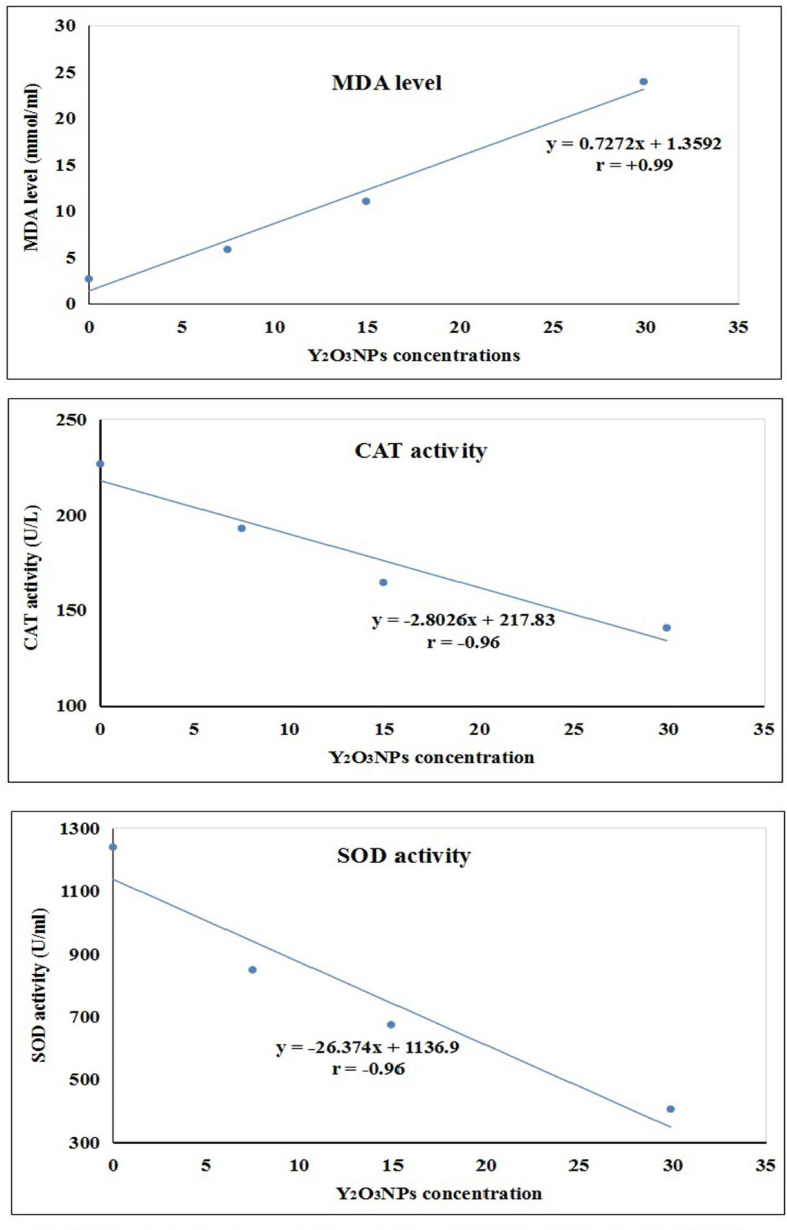
Regression lines and correlation coefficient between Y 2 O 3 NPs concentrations (7.47. 14.94 and 28.89 µg/ml) and the measured MDA level, CAT activity and SOD activity using suitable biochemical kits.

Consistency with ROS generation results, biochemical measurement of oxidative stress markers demonstrated remarkable induction of oxidative stress within A-431 cancer cells by Y_2_O_3_NPs treatment in a concentration-dependent manner compared to both control and doxorubicin-treated A-431 cancer cells (Table 4). This was manifested by the concentration-dependent statistical significant elevation in the MDA level, a by-product lipid peroxidation, concurrently with the significant decreases in the activities of the antioxidant CAT and SOD enzymes noticed after 72 h of A-431 cancer cells treatment with Y_2_O_3_NPs various concentrations (7.47, 14.98 and 29.89 µg/ml) compared to their values in both the control and doxorubicin-treated A-431 cancer cells (Table 4). Regression analysis also demonstrated the strong correlations between the tested Y_2_O_3_NPs concentrations and the measured oxidative stress parameters: MDA level was strongly positively correlated (r = + 0.99) with various Y_2_O_3_NPs concentrations, while of CAT and SOD antioxidant activities were strongly negatively correlated (*r*= -0.96) with different Y_2_O_3_NPs concentrations (Fig. 8).

**Table 4:** Level of MDA and activity of antioxidant CAT and SOD enzymes in the control (untreated) and doxorubicin- or Y_2_O_3_NPs-treated A-431 cancer cells.

Treatment	Concentration (µg/ml)	MDA level (mmol/ml)	CAT activity (U/L)	SOD activity (U/ml)
**Control cells**	**0.00 µg/ml**	**2.67 ± 0.09** ^a^	**226.55 ± 3.75** ^a^	**1239.67 ± 34.43** ^a^
**Doxorubicin**	**2 µg/ml /ml**	**5.15 ± 0.10** ^b^	**186.97 ± 2.14** ^b^	**799.07 ± 11.34** ^b^
**Y**_**2**_ **O**_**3**_**NPs**	**1/4 IC50** **(7.47 µg/ml)**	**5.84 ± 0.06** ^**b**^	**192.88 ± 4.40** ^**b**^	**848.79 ± 48.89** ^**b**^
	**1/2 IC50** **(14.94 µg/ml)**	**11.03 ± 1.46** ^**c**^	**164.54 ± 5.03** ^**c**^	**674.12 ± 42.97** ^**c**^
	**IC50** **(29.89 µg/ml)**	**23.93 ± 2.10** ^**d**^	**140.77 ± 6.65** ^**d**^	**405.67 ± 39.53** ^**d**^
**ANOVA**		**F = 165.20 < 0.001** *p*	**F = 144.01 < 0.001** *p*	**F = 198.75 < 0.001** *p*

• **Results are expressed as mean ± SD**.

• **Results were analyzed using One Way Analysis of Variance (ANOVA) followed by Duncan’s test.**

• **Different superscript letters indicates statistical significant difference among the compared untreated control and treated cells at < 0.001.***p*

• **F value is the ratio of between and within group variances**.

### Integrity of mitochondrial membrane potential in A-431 cells

**Fig. 9:**
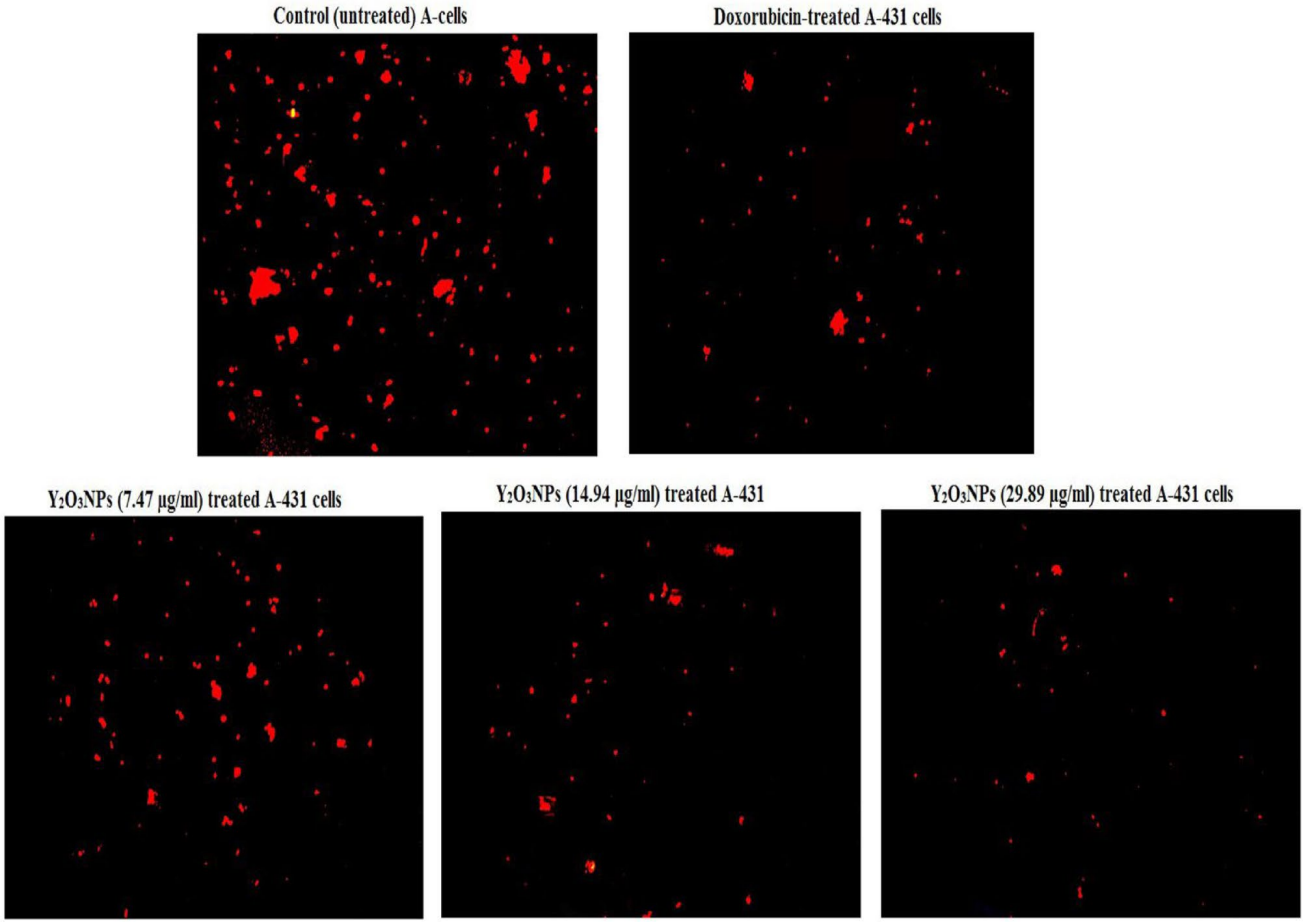
Integrity of mitochondrial membrane potential studied using Rhodamine-123 dye in the control untreated A-431 cells and A-431 cells treated with doxorubicin- or Y 2 O 3 NPs (7.47. 14.94 and 28.89 µg/ml) for 72 h.

As displayed in Fig. 9 and Table 3a dramatic concentration-dependent reduction in the integrity of mitochondrial membrane potential occurred upon treatment of A-431 cancer cells with Y_2_O_3_NPs concentrations of 7.47, 14.98 and 29.89 µg/ml, as shown by the high reductions seen in the fluorescent light intensity emitted by Y_2_O_3_NPs-treated A-431 cancer cells compared to the light emitted by either control (untreated) or doxorubicin-treated A-431 cancer cells (Fig. 9).

### Expression of apoptotic and mitochondrial genes in A-431 cells

**Fig. 10:**
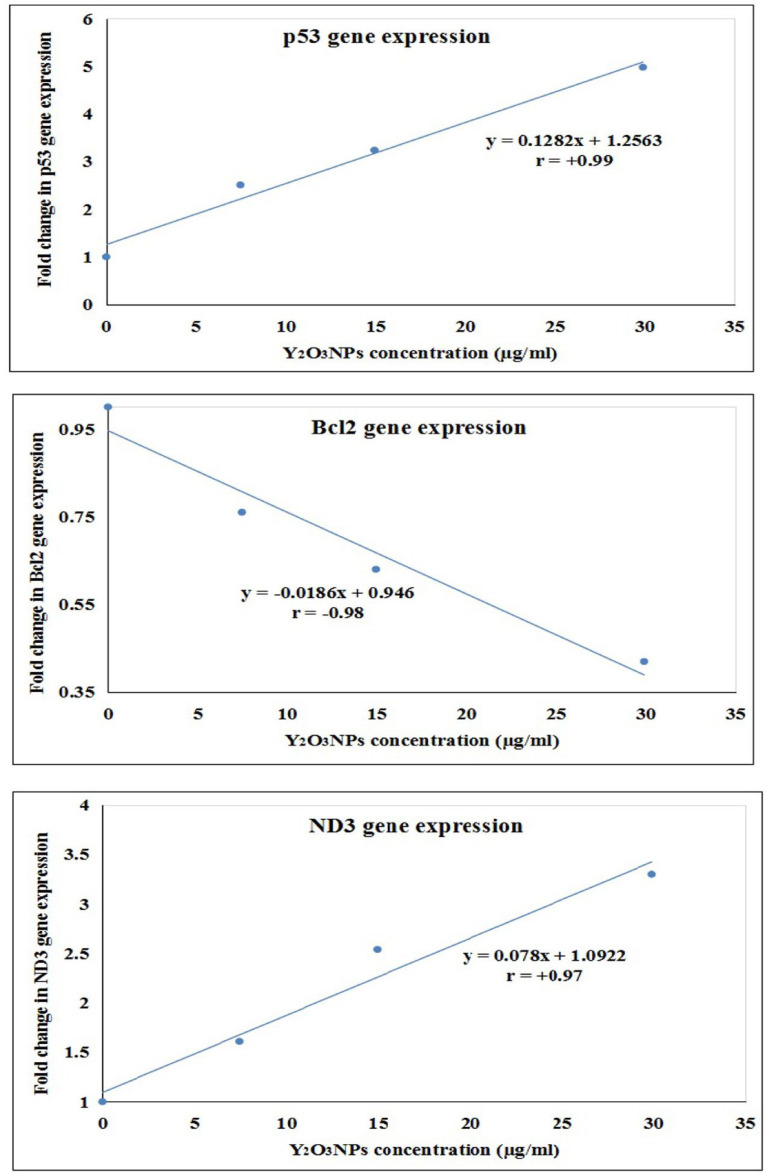
Regression lines and correlation coefficient between Y2O3NPs concentrations (7.47. 14.94 and 28.89 µg/ml) and the expression level of p53, Bcl2 and ND3 genes measured using qRT-PCR.

Interpretation of qRT-PCR results revealed that treatment of A-431 cancer cells with Y_2_O_3_NPs at concentrations of 7.47, 14.98 and 29.89 µg/ml significantly upregulated the expression level of apoptotic p53 and mitochondrial ND3 genes, while significantly downregulated the anti-apoptotic Bcl2 gene expression in a concentration-dependent manner compared to the expression level in control (untreated) A-431 cells (Table 5). Meanwhile, exposure of A-431 cancer cells to Y_2_O_3_NPs also significantly elevated the expression level of apoptotic p53 and mitochondrial ND3 genes compared to the elevations induced by doxorubicin treatment as depicted in Table 5. The results of the regression analysis shown in Fig. 10 evidenced the strong dependence of gene expression on Y_2_O_3_NPs concentrations where a strong negative correlation (*r*= -0.98) was detected between Bcl2 gene expression and the concentrations of Y_2_O_3_NPs tested, and a strong positive correlation was reported between Y_2_O_3_NPs concentrations and the expression level of p53 (r = + 0.99) and ND3 (r = + 0.97) genes.

**Table 5:** Expression level of apoptotic (p53 and Bcl2) and mitochondrial ND3 genes in the in the control (untreated) and doxorubicin- or Y_2_O_3_NPs-treated A-431 cancer cells.

Treatment	Concentration (µg/ml)	Fold change in the expression level of		
		p53 gene	Bcl2 gene	ND3 gene
**Untreated cells**	**0.00 µg/ml**	**1.00 ± 0.00** ^a^	**1.00 ± 0.00** ^a^	**1.00 ± 0.00** ^a^
**Doxorubicin**	**2 µg/ml**	**3.35 ± 0.17** ^**b**^	**0.49 ± 0.06** ^**b**^	**1.86 ± 0.16** ^**b**^
**Y**_**2**_ **O**_**3**_**NPs**	**1/4 IC50** **(7.47 µg/ml)**	**2.51 ± 0.03** ^**c**^	**0.76 ± 0.02** ^**c**^	**1.61 ± 0.14** ^**b**^
	**1/2 IC50** **(14.94 µg/ml)**	**3.24 ± 0.11** ^**b**^	**0.63 ± 0.03** ^**d**^	**2.54 ± 0.18** ^**c**^
	**IC50** **(29.89 µg/ml)**	**4.98 ± 0.46** ^**d**^	**0.42 ± 0.11** ^**b**^	**3.30 ± 0.14** ^**d**^
**ANOVA**		**F = 124.77 < 0.001** *p*	**F = 44.83 < 0.001** *p*	**F = 117.09***p*< 0.001

•**Results are expressed as mean ± SD**.

•**Results were analyzed using One Way Analysis of Variance (ANOVA) followed by Duncan’s test.**

•**Different superscript letters indicate statistical significant difference among the compared untreated control and treated cells at***p*< 0.001.

• **F value is the ratio of between and within group variances**.

## Discussion

The lack of specificity and negative human health side effects of commercially used drugs for epidermoid skin cancer treatment alongside with the recently discovered promising selective and strong cytotoxicity of Y_2_O_3_NPs against highly aggressive triple negative breast and prostate cancer cells, necessitated the estimation of the undiscovered impact of Y_2_O_3_NPs exposure on epidermoid skin cancer. Therefore, this study was conducted to estimate the influence of Y_2_O_3_NPs treatment on the viability of normal HSF and epidermoid skin cancer A-431 cells, along with their impact on genomic DNA and mitochondrial integrity, ROS generation, and the induction of oxidative stress and apoptosis in A-431 cells to better understand the mechanism of action of Y_2_O_3_NPs.

The SRB cytotoxicity assay results demonstrated the potent selective cytotoxicity of Y_2_O_3_NPs against A-431 epidermoid cancer cells through the remarkable ability of Y_2_O_3_NPs to preferentially induce marked concentration dependent cell death in A-431 cancer cells while sparing normal HSF cells with a high selectivity index value of 14.76. These results supported recent studies reporting that Y_2_O_3_NPs could preferentially target human triple negative breast cancer, malignant glinoma and prostate cancer cells, while having minimal impact on the viability of human normal retinal RPE1 cells and dermal fibroblast HDF cells^8,9^.

Induction of over-ROS production and oxidative stress is one of the plausible mechanisms for the selective cytotoxicity of Y_2_O_3_NPs in cancer cells because elevated ROS levels disrupt the cellular balance between oxidant and antioxidants, causing damage to cellular macromolecules, including lipids, carbohydrates, proteins, and DNA, with increasing the susceptibility of cancer cells to oxidative stress-induced damage compared to normal cells^22^. Consequently, our findings on the potent selective cytotoxicity of Y_2_O_3_NPs against A-431 epidermoid skin cancer cells may be due to the remarkable excess ROS generation and oxidative stress induction noticed in Y_2_O_3_NPs-treated A-431 cancer cells. Consistent with previous findings, excessive production of harmful ROS attacks and damages cellular proteins, lipids and DNA while inhibiting the antioxidant defense system leading to oxidative stress and apoptosis^23–25^. The induction of oxidative stress in A-431 cancer cells treated with Y_2_O_3_NPs was reflected by the significant concentration-dependent elevations in the level of MDA, a by-product of lipid peroxidation, along with significant decreases in the activities of the antioxidant enzymes CAT and SOD reported after 72 h of A-431 cancer cells exposure to tested concentrations of Y_2_O_3_NPs.

Excessive ROS generation and oxidative stress induction within Y_2_O_3_NPs-treated A-431 cancer cells thus exhausted cells and triggered apoptosis. Apoptosis induction in the A-431 cancer cells treated with Y_2_O_3_NPs was proved from the findings of Chromatin diffusion assay that sensitively revealed the fragmentation and diffusion of chromatin providing clear morphological evidence for A-431 cells apoptosis after exposure to Y_2_O_3_NPs for 72 h constituently with the recent studies^13,26^ Chromatin diffusion assay is a sensitive and informative method for detecting apoptosis, offering valuable insights into chromatin changes during programmed cell death and providing direct morphological evidence of apoptosis.

Excessive generation and accumulation of ROS beyond the basal threshold can induce apoptosis in cancer cells by causing mitochondrial damage. This damage affects the mitochondrial permeability transition pore and leads to mitochondrial membrane depolarization^27,28^. In A-431 cancer cells exposed to Y_2_O_3_NPs for 72 h, this mitochondrial damage was evident from the dramatic loss of mitochondrial membrane potential.

Consequently, Y_2_O_3_NPs induced apoptosis in A-431 cancer cells can be attributed to the demonstrated ROS associated mitochondrial damage that disrupts the permeability and polarization of mitochondrial membrane causing the release of apoptosis inducing factors in the cytoplasm^29^. One such factor is the mitochondrial ND3 gene that encodes the mitochondrial respiratory chain complex I subunit, and triggers apoptosis releasing pro-apoptotic factors into the cytosol^30,31^. Consistency with previous studies, the significant upregulation of the mitochondrial ND3 gene detected in Y_2_O_3_NPs treated A-431 cancer cells forced them to undergo apoptosis because this overexpression led to a shift from anaerobic glycolysis to aerobic respiration, depriving the cancer cells of glucose and thus inhibiting their proliferation^32^.

The ROS-mediated mitochondrial pathway of apoptosis in Y_2_O_3_NPs treated A-431 cancer cells was further confirmed through the noticed significant increases in the expression of the apoptotic gene p53 in conjunction with significant decreases in the expression of the anti-apoptotic gene Bcl2 because overexpression of the tumor suppressor p53 gene also stimulate apoptosis through direct interaction with the anti-apoptotic Bcl-2 genes leading to mitochondrial membrane depolarization and increasing the release of apoptotic signals. Specifically, p53 promotes p53 upregulated modulator of apoptosis (Puma) and Bcl2 homology domain 3 (BH3) (Noxa), the two core proteins of the Bcl2 family since Puma enhances Bcl-2-associated X protein (BAX) and Noxa supports p53-mediated apoptotic signaling^33,34^.

Consequently, this study demonstrated for the first time the selective strong cytotoxic efficacy of Y_2_O_3_NPs through over ROS generation that induced oxidative stress and mitochondrial mediated apoptosis of A-431 cancer cells as displayed in the provided schematic diagram. However, some limitations could be associated with this study such as using only one normal and one cancer cell line.

## Conclusion

Based on the findings discussed above, Y_2_O_3_NPs exhibited a promising selective and strong cytotoxic effect against A-431 epidermoid skin cancer cells in a concentration-dependent manner. The mechanism behind this strong cytotoxicity appears to be linked to excessive generation of ROS, which significantly damages mitochondria and alters the expression levels of key apoptotic factors, including p53, the anti-apoptotic protein Bcl2, and mitochondrial ND3 genes. This cascade of events suggests the induction of the ROS-mediated mitochondrial pathway of apoptosis. Meanwhile, Y_2_O_3_NPs did not show significant toxicity to normal HSF cells, highlighting their potential therapeutic efficacy against epidermoid skin cancer. To fully explore the applicability of Y_2_O_3_NPs in the treatment of skin cancer, further in vitro and in vivo studies are highly recommended.

## Data Availability

The datasets used and/or analyzed during the current study are available from the corresponding author on reasonable request.
